# Drug Screening Identifies Sigma-1-Receptor as a Target for the Therapy of VWM Leukodystrophy

**DOI:** 10.3389/fnmol.2018.00336

**Published:** 2018-09-18

**Authors:** Andrea Atzmon, Melisa Herrero, Reut Sharet-Eshed, Yocheved Gilad, Hanoch Senderowitz, Orna Elroy-Stein

**Affiliations:** ^1^The School for Molecular Cell Biology and Biotechnology, George S. Wise Faculty of Life Sciences, Tel Aviv University, Tel Aviv, Israel; ^2^Department of Chemistry, Bar-Ilan University, Ramat-Gan, Israel; ^3^Sagol School of Neuroscience, Tel Aviv University, Tel Aviv, Israel

**Keywords:** leukodystrophy, drug screening, vanishing white matter (VWM), mitochondria dysfunction, eIF2B, Sigma-1-Receptor (S1R), agonist, drug-like compounds

## Abstract

Vanishing white matter (VWM) disease is an autosomal genetic leukodystrophy caused by mutations in subunits of eukaryotic translation initiation factor 2B (eIF2B). The clinical symptoms exhibit progressive loss of white matter in both hemispheres of the brain, accompanied by motor functions deterioration, neurological deficits, and early death. To date there is no treatment for VWM disease. The aim of this work was to expedite rational development of a therapeutic opportunity. Our approach was to design a computer-aided strategy for an efficient and reliable screening of drug-like molecules; and to use primary cultures of fibroblasts isolated from the Eif2b5^R132H/R132H^ VWM mouse model for screening. The abnormal mitochondria content phenotype of the mutant cells was chosen as a read-out for a simple cell-based fluorescent assay to assess the effect of the tested compounds. We obtained a hit rate of 0.04% (20 hits out of 50,000 compounds from the selected library). All primary hits decreased mitochondria content and brought it closer to WT levels. Structural similarities between our primary hits and other compounds with known targets allowed the identification of three putative cellular pathways/targets: 11β-hydroxysteroid dehydrogenase type 1, Sonic hedgehog (Shh), and Sigma-1-Receptor (S1R). In addition to initial experimental indication of Shh pathway impairment in VWM mouse brains, the current study provides evidence that S1R is a relevant target for pharmaceutical intervention for potential treatment of the disease. Specifically, we found lower expression level of S1R protein in fibroblasts, astrocytes, and whole brains isolated from Eif2b5^R132H/R132H^ compared to WT mice, and confirmed that one of the hits is a direct binder of S1R, acting as agonist. Furthermore, we provide evidence that treatment of mutant mouse fibroblasts and astrocytes with various S1R agonists corrects the functional impairments of their mitochondria and prevents their need to increase their mitochondria content for compensation purposes. Moreover, S1R activation enhances the survival rate of mutant cells under ER stress conditions, bringing it to WT levels. This study marks S1R as a target for drug development toward treatment of VWM disease. Moreover, it further establishes the important connection between white matter well-being and S1R-mediated proper mitochondria/ER function.

## Introduction

Vanishing white matter (VWM) disease, also termed Childhood ataxia with CNS hypomyelination (CACH), is a genetic autosomal recessive leukodystrophy characterized by progressive loss of white matter in both hemispheres of the brain. The consequent axonal degeneration results in progressive impairments of neurologic functions, leading to complete paralysis and early death (Schiffmann and Elroy-Stein, 2006; Elroy-Stein and Schiffmann, 2015). Disease onset and clinical symptoms refer to congenital, classical, and adult forms. The congenital form is extremely rare; the classical form refers to disease onset at early childhood and death around late teens, with <1000 known patients in the world; the adult form which seems increasingly common refers to a mild version of clinical symptoms and their slow progression. An important feature of all forms of this genetic orphan disease is the deterioration of clinical symptoms upon exposure to physiological and environmental stressors (Fogli and Boespflug-Tanguy, 2006).

Vanishing white matter disease is caused by partial-loss-of-function mutations in any of the five genes encoding the subunits of eIF2B (Leegwater et al., 2001). eIF2B is a master regulator of mRNA translation at the hub of the integrated stress response (ISR) (Pavitt, 2018). The five subunits of eIF2B, which assemble into a decameric (αβδγε)_2_ complex (Kashiwagi et al., 2016), recycle GDP-bound eIF2 back to its active GTP-bound form, under normal conditions, while it fails to do so when eIF2α is phosphorylated upon stress. Hypo-active eIF2B renders mutant cells hyper-sensitive to endoplasmic reticulum (ER) stress (Kantor et al., 2005).

To further study the disease etiology, we have previously developed the Eif2b5^R132H/R132H^ mouse strain which is homozygous for one of the VWM mutations. This mouse model exhibits ∼20% decrease in eIF2B enzymatic activity in the brain, which is associated with white matter deficits and mild impairment of motor functions. Eif2b5^R132H/R132H^ mice provided fundamental insights related to the effect of eIF2B mutations, including delayed postnatal brain development, failure to overcome cuprizone-induced demyelination, abnormal glial cell abundance, increased abundance of demyelinated axons, and axons ensheathed with split and damaged myelin (Geva et al., 2010). Astrocytes are considered central in the patho-mechanism of the disease (Dooves et al., 2016), yet eIF2B mutations lead to cellular phenotype impairments also in microglia, associated with poor cerebral inflammatory response upon insults (Cabilly et al., 2012). At the molecular level, we have demonstrated delayed waves of gene expression, unbalanced expression of unfolded proteins response (UPR)-related genes, impaired oxidative respiration, and increased mitochondrial content to compensate for energetic requirements (Marom et al., 2011; Gat-Viks et al., 2015; Raini et al., 2017).

To date there is no treatment for VWM disease. Earlier attempts to identify eIF2/eIF2B modifying compounds in the context of ISR or eIF2B mutations involved a recombinant yeast-based assay for high-throughput screening (Motlekar et al., 2009). A later use of recombinant stress-reporting assay in mammalian cells identified a molecule, known as ISRIB (Sidrauski et al., 2013). ISRIB stabilizes the decameric form of eIF2B and restores the residual catalytic activity of some eIF2B mutant complexes to wild-type (WT) levels, while rendering it insensitive to the inhibitory effect of eIF2α phosphorylation (Wong et al., 2018; Zyryanova et al., 2018). In the current study, we undertook a different approach by using the defective mitochondrial phenotype of primary fibroblasts isolated from Eif2b5^R132H/R132H^ mice. We used this cellular phenotype to develop a cellular assay for screening of compounds that decrease mitochondria content. By comparing the structure of 19 relevant hits to that of compounds with known biological targets, 6 were mapped to three putative cellular targets/pathways: Sonic hedgehog (Shh), Sigma-1-Receptor (S1R), and 11β-hydroxysteroid dehydrogenase type 1 (11β-HSD1). We experimentally confirmed that four compounds act as antagonists/agonists of the Shh signaling pathway and provided an initial indication that it is impaired in VWM disease. Moreover, we demonstrated lower expression level of S1R protein in fibroblasts, astrocytes, and whole brains isolated from Eif2b5^R132H/R132H^ mice. Importantly, the current study clearly shows that treatment of mutant MEFs and astrocytes with S1R agonists corrects their mitochondrial functional impairments and enhances their survival rate under ER-stress conditions. Therefore, this study marks S1R as a highly relevant target for pharmacological intervention for the treatment of VWM disease.

## Materials and Methods

### Mice and Cells

Wild-type (C57BL strain) and Eif2b5^R132H/R132H^ (Mut; mutant) mice of both sexes were bred and housed in Tel Aviv University animal facility with 14/10 h light/dark cycle in groups of four animals per cage in individually ventilated cages (Lab Products Inc., Seaford, DE, United States) supplemented with autoclaved wood chips. Animals fed with autoclaved rodent pellet (Koffolk 19-510; Koffolk Ltd., Petach Tikva, Israel) and sterile water *ad libitum*. All experimental procedures were approved by the Tel Aviv University Animal Care Committee according to national guidelines (permits #L-15-037 and #04-12-27). Breeding and genotyping was performed as previously described (Geva et al., 2010). Each generation was established by back cross of homozygous Mut with WT C57BL/6J (Harlan Labs, Jerusalem, Israel) to prevent genetic drift. Primary cultures of MEFs (from E14 embryos) and astrocytes (from P0-P2 newborns) were isolated and used as previously described (Raini et al., 2017).

Sonic hedgehog-light2 cells (Taipale et al., 2000) were maintained in DMEM supplemented with 10% fetal bovine serum, 100 U/ml penicillin, 0.1 mg/ml streptomycin, 2 mM L-glutamine, 400 μg/ml G418 (A.G. Scientific), and 200 μg/ml Zeocin (InvivoGen).

### RNA and Protein Extractions From Brains

Brains were removed from p14, P18, and P21 mice. Cerebrums were flash frozen in liquid nitrogen and kept in -80°C until use. RNA was extracted from left hemispheres using RiboEX (GeneAll). Proteins were extracted from left hemispheres by sonication in 500 μl per hemisphere of lysis buffer containing 1% triton, 0.5% NaDOC, 0.1% SDS, 50 mM Tris pH 8, 100 mM NaCl, 10 mM β-glycerophosphate, 5 mM NaF, 1 mM DTT, 1 mM vanadate, and EDTA-free complete^TM^ protease inhibitor cocktail (#11-836-170-001; ROCHE). After spinning for 15 min, 13,000 rpm, 4°C, the supernatant was analyzed for total concentration using BCA protein assay kit (#23227 Pierce).

### Compounds

The compounds selected from DIVERSet^TM^-EXP library and H8 analogs were purchased from ChemBridge (San Diego, CA, United States). SAG (#4366), cyclopamine (#1623), pre-084 (#0589), and NE-100 (#3133) were from Tocris Bioscience. Pridopidine (#M326195) was from Toronto Research Chemicals (Toronto, ON, Canada), Tunicamycin (#T7765) from Sigma. All compounds were dissolved in DMSO, cyclopamine was dissolved in ethanol.

### Selection and Clustering of Screening Library

The algorithm for the selection of optimal screening libraries is described in detail in Gilad et al. (2015) and was followed with no modifications. Each of the 50,000 compounds comprising the DIVERSet^TM^-EXP library was characterized by the ECFP6 fingerprints as implemented in BIOVIA’s Discovery Studio Version 3.5 and clustered into 500 clusters using the Hierarchical clustering as implemented in Schrodinger’s Canvas (Canvas, Schrödinger, LLC, New York, NY, 2018, United States). From each cluster, a compound closest to its center was selected for biological evaluation.

### Docking and *in vitro* Confirmation of S1R Binding

All calculations were performed in BIOVIA’s Discovery Studio Version 3.5. The crystal structure of S1R in complex with *N*-(1-benzylpiperidin-4-yl)-4-iodobenzamide (code 5HK2 in PDB protein data base) was retrieved from the PDB. Prior to docking, the protein structure was prepared using the prepare protein protocol. Docking was performed with the CDOCKER program using default parameters. Confirmation of S1R binding was performed by *in vitro* competitive displacement-binding assay using the known S1R binder [^3^H]-haloperidol (Ganapathy et al., 1999). The test was executed by Eurofin Central Laboratory Inc.

### Image-Based Single Cell Analysis

MEFs were seeded on 1% gelatin-coated 96-well plate at a density of 5000 cells per well. Twenty-four hours post-plating cells were incubated with the tested compounds for 24 h. Several DMSO-treated cells (control) were included in each plate at different locations. The cells were then stained by addition of fluorogenic dyes for further incubation for 30 min at 37°C. Hoechst 33258 (#861405; Sigma-Aldrich) and JC-1 (#T4069; Sigma-Aldrich) were used at final concentration of 2 μg/ml; CellTrace CFSE (#C345545; Molecular Probes), and CellROX Deep Red (#C10422; Molecular Probes) at final concentration of 5 μM. CellROX was used together with Hoechst and CFSE; JC1 was used together with Hoechst. Cells were washed with Hank’s balanced salt solution (HBSS) used for images acquisition by IN Cell Analyzer 2000 (GE Healthcare, Pittsburgh, PA, United States). IN Cell Developer Toolbox 1.9.1 software (GE Healthcare, Pittsburgh, PA, United States) served for analysis. Analysis included cells segmentation using Hoechst and/or CFSE signals. For analysis of JC1 staining, integrated intensity of green and red emissions served for detection of damaged and intact mitochondria, respectively.

### Cell Survival Assay

Cells were seeded on 96-well plate at a density of 5000 cells per well. Astrocytes were seeded following coating with 0.001% PDL. Twenty-four hours post-plating cells were incubated with the tested compounds for 24 h followed by staining with 0.1% crystal violet/4% formaldehyde/1% ethanol as described in Heiss et al. (2014).

### Quantification of Gli1 mRNA

Total RNA was subjected to reverse transcription using qscript cDNA synthesis kit (#95047 Quanta Biosciences) and subjected to qPCR analysis using SYBR-Green (PerfeCTa^®^ SYBR^®^ Green FastMix^®^, ROX^TM^; #95073; Quanta Biosciences) and the following oligonucleotide primers: Gli1 Fwd 5′-CCCATAGGGTCTCGGGTCTCAAAC-3′ and Gli1 Rev 5′-GGAGGACCTGCGGCTGACTGTGTAA-3′ for Gli1 mRNA amplification and Gapdh Fwd 5′-TGGCAAAGTGGAGATTGTTGCC-3′ and Gapdh REV 5′-AAGATGGTGATGGGCTTCCCG-3′ for Gapdh mRNA as an internal control. Equal amounts of RNA were used and reactions were carried out for 40 cycles in StepOne Real-time PCR apparatus (Applied Biosystems). Average relative quantity (RQ) was calculated by the ΔΔCt method.

### Luciferase Activity Assay

Sonic hedgehog-LIGHT2 cells were seeded at a density of 10,000 cells per well of a 96-well plate in growing medium. Twenty-four hours post-plating cells were incubated for 24 h with the tested compounds in low serum media (0.5%) without G418 and Zeocin. Following lysis, Firefly and Renilla luminescence was measured using the Dual Luciferase assay kit (Promega) and a Veritas microplate luminometer (Turner Biosystems).

### Western Blot Analysis

10^5^ Astrocytes or 1.5 ^∗^ 10^5^ MEFs were seeded per well of a six-well plate and cultured for 3 or 2 days, respectively. After one wash with PBS, 100 μl of lysis buffer containing 1.6% SDS, 80 mM DTT, 8% glycerol, 64 mM Tris pH 6.8 were applied directly on the cell monolayer followed by extract collection. Forty microliters per lane of MEFs total cell extract, 10 μl per lane of astrocytes total cell extract, or 30 μg total protein per lane of brain protein extract were separated by 15% SDS–PAGE followed by immunoblot analysis using antibodies specific for Sig1R (Santa Cruz #137075) and SDHB (Abcam #ab14714). The enhanced chemiluminescence signal was captured using a AI600 Imager (Amersham) and quantified by ImageQuant TL (GE Healthcare, Pittsburgh, PA, United States).

### mtDNA Quantification

Total DNA was extracted using GenElute^TM^ (#G1N350; Sigma-Aldrich) and used for real-time SYBR-Green-based PCR reactions (PerfeCTa^®^ SYBR^®^ Green FastMix^®^, ROX^TM^; #95073; Quanta Biosciences) using 40 cycles in StepOne Real-time PCR apparatus (Applied Biosystems). The following oligonucleotide primers were used: 5′-ACCGCGGTCATACGATTAAC-3′ and5′-CCCAGTTTGGGTCTTAGCTG-3′ (12SrRNAmt FWD and 12SrRNAmt REV, respectively, to amplify the gene encoding mitochondrial 12S rRNA); and 5′-CGCGGTTCTATTTTGTTGGT-3′ and 5′-AGTCGGCATCGTTTATGGTC-3′ (18SrRNAn FWD and 18SrRNAn REV, respectively, to amplify the nuclear gene encoding 18S rRNA). Average RQ of mtDNA per nucDNA values were calculated and normalized to the experimental control value.

### TMRE Staining

MEFs were seeded at a density of 4 ^∗^ 10^4^ cells per well of a 24-well plate. Twenty-four hours post-plating the cells were incubated with the tested compounds for 6 h followed by staining with 200 nM tetra-methyl-rhodamine-ethyl esterperchlorate (TMRE) (Abcam) for 30 min at 37°C. Cells were removed by trypsinization, washed, and resuspended in PBS. 5–10 ^∗^ 10^3^ cells were analyzed by Stratedigm S1000EXi cell sorter and FlowJo software (FLOWJO, LLC, Ashland, OR, United States).

### Oxygen Consumption

Oxygen consumption was measured in primary MEFs and astrocytes using a XF96 extracellular Flux Analyzer (Seahorse Bioscience, Billerica, MA, United States) as described (Invernizzi et al., 2012). Primary MEFs or astrocytes were seeded in a XF96-well cell culture microplate at a density of 5000 cells/well and incubated for 24 or 72 h, respectively, in 80 μL of MEF medium or astrocytes medium (#1801; Sciencell Research Labs), at 37°C in 5% CO_2_ atmosphere prior to analysis. The Mito Stress Test Kit (Agilent Technologies, Santa Clara, CA, United States) was used to measure oxygen consumption rate (OCR) as detailed in Raini et al. (2017). ATP-linked respiration and maximal capacity respiration were determined as recommended by the supplier. Oligomycin (Sigma, #O4876) was used at concentrations of 1 μM for MEFs and 2 μM for astrocytes; FCCP (#C2920, Sigma) at 1.5 μM, antimycin A (#A8674, Sigma) at 0.5 μM for MEFs and 1 μM for astrocytes; and rotenone (#R8875, Sigma) at 0.5 μM for MEFs and 1 μM for astrocytes. MEFs data were normalized to mtDNA/nucDNA content. Astrocytes data were normalized to cell number, obtained by Crystal Violet staining (Heiss et al., 2014).

### Statistics

For all comparisons, Student’s *t*-test was performed using more than or equal to three independent biological repeats for each group (methodological selection of sample size was not applied).

## Results

### Selection of Library and Screening of Compounds

A computational workflow for rational selection of an optimal screening library (Gilad et al., 2015) was applied to the analysis of nine commercially available libraries containing ∼380,000 compounds. We ranked the libraries based on three criteria: (1) Absorption, Distribution, Metabolism, Excretion, and Toxicity (commonly abbreviated ADME/T) profiling. This pharmacokinetic profile is of paramount importance in determining the fate of a compound within the body and consequently its drug-like properties. As part of this profiling, we developed a new Quantitative Structure Activity Relationship (QSAR) model for the prediction of blood brain barrier permeability. Briefly, QSAR models are mathematical equations correlating activities with structure-based descriptors for a set of compounds; (2) internal diversity; and (3) similarity to the known active compound Guanabenz. We included the third criteria because Guanabenz was proposed to inhibit PPP1R15A, a stress-induced subunit of protein phosphatase 1 (PP1), responsible for eIF2α dephosphorylation (Tsaytler et al., 2011). This information was relevant for us since a genetic knockout of PPP1R15A gene corrected the VWM phenotype of Eif2b5^R132H/R132H^ mice (unpublished data). Importantly, by using non-phosphorylatable eIF2α (eIF2α*^*S51A*^*) cells, a recent study challenged the fact that Guanabenz interferes with PPP1R15A-mediated eIF2α-P dephosphorylation (Crespillo-Casado et al., 2017). However, Bergmann glia pathology and cerebellar myelin pathology improved with Guanabenz treatment in mice (Dooves et al., 2018).

All three criteria were combined into a single library score using a consensus approach while assigning equal weights to each. The rankings based on the individual criteria and the consensus rankings are presented in **Table 1**. These results indicate that the DIVERSet^TM^-EXP library by *Chembridge* was the best screening library for the current project.

**Table 1:** Library ranking.

Library	Size	ADME/T ranking	Diversity ranking	Similarity ranking	Final rank
Elite Libraries	70,114	2	8	8	8
Platinum Collection	113,962	6	7	5	8
DIVERSet^TM^-CL	50,000	2	4	7	5
DIVERSet^TM^-EXP	50,000	1	5	1	1
Drug-Like Set	20,160	3	2	3	2
Pharmacological Diversity Set	10,240	3	6	6	6
Maybridge Screening Collection	54,318	4	3	2	3
Prestwic Chemical Library^®^	1,280	7	1	9	7
MSII Full Library	10,000	5	2	4	4

For screening, we took advantage of the differential cellular phenotypic characteristics of primary mouse embryonic fibroblasts (MEFs) isolated from Eif2b5^R132H/R132H^ mouse model (Geva et al., 2010) and based the assay on their abnormally high mitochondria content. This approach was chosen because our previous study have shown that increased mitochondrial biogenesis in eIF2B-mutant MEFs is a compensatory response to overcome compromised oxidative phosphorylation (Raini et al., 2017). We hypothesized that incubation of mutant cells with a compound leading to enhanced mitochondrial function will alleviate their need to increase mitochondria content. Since our cellular assay relies on small differences in mitochondrial function, it became apparent that medium throughput screening employing several repeats with several batches of cells is likely to give much better results than high-throughput screening. This required reducing the number of compounds tested at each cycle. We anticipated that experimental testing of a subset of compounds, which well represents the entire parent library (i.e., a representative subset), should provide a similar amount of information as would be provided by testing the entire library. This hypothesis stems from the similar property principle, which states that similar compounds have similar properties. We therefore clustered the DIVERSet^TM^-EXP library into 500 clusters and selected a single representative from each cluster, closest to the cluster center. Following biological testing, active hits easily traced back to their parent clusters. Testing additional members of “active clusters” provided valuable SAR information.

**FIGURE 1:**
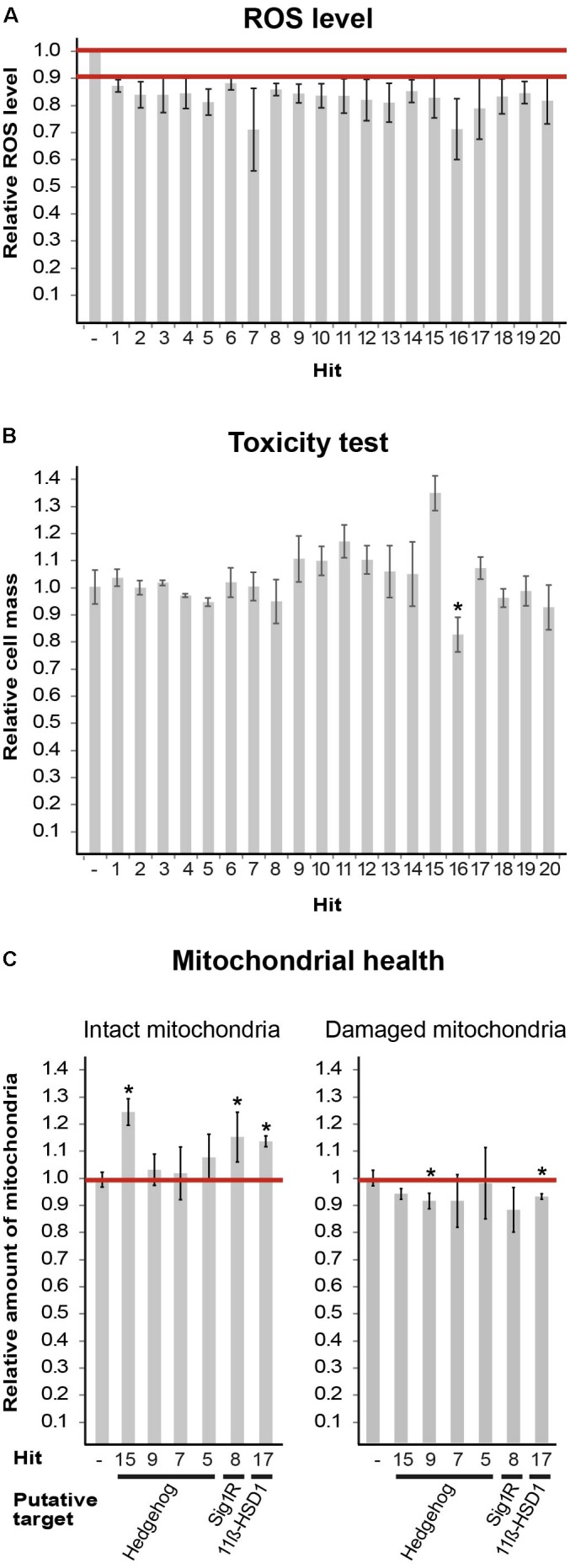
Hits obtained from DIVERSet^TM^-EXP library. **(A)** Mut MEFs treated with the indicated compounds at 10 μM for 24 h followed by staining with fluorogenic dyes and image-based single-cell analysis. Shown is average effect on ROS level of 20 final hits (**Table 2**) from at least three-independent experiments ± SEM. All values are statistically significant, *p* < 0.02. The extreme SEM points of all values are below the 90% cut-off; therefore, the actual average reduction is between 12 and 29%. **(B)** Mut MEFs were incubated or not for 24 h with 10 μM of the indicated compound (**Table 2**) followed by crystal violet staining. Shown is average absorbance at OD = 570 nm as a proxy of cell mass, compared to cells in the absence of any compound, of three independent experiments ± SEM, ^∗^*p* = 0.0003. **(C)** Mut MEFs were incubated or not for 24 h with 10 μM of the indicated compound (**Table 2**) followed by JC1 and Hoechst staining. Image-based single-cell analysis using JC1 red and green emission employed to detect intact and damaged mitochondria, respectively. Values of untreated cells were set as 1. Shown are average values of at least three independent experiments for each parameter compared to its matching control ± SEM. ^∗^*p* < 0.05. Putative targets are indicated.)

To assay the compounds, we used *cellROX*, a reactive oxygen species (ROS) fluorescent detector and a preferred *in situ* mitochondria content detector, for single cell-based imaging analysis as detailed previously (Raini et al., 2017). Briefly, the median *cellROX* integrated intensity of non-treated cells was set as a threshold to define the population above it, as “high-ROS cells.” This fraction, being 50% in non-treated cells, was set as 1. We expected it to be <1 if a compound acts via one of numerous possible mechanisms to decrease ROS levels. A compound was considered a “hit” if it was able to decrease the size of “high-ROS cells” population to 0.9 or below, in at least three independent experiments. The first screening round identified eight hits, each representing a different cluster. In the second round, we screened 437 compounds that were included in the eight relevant clusters and identified 20 hits, termed H1–H20 (**Figure 1A**; see chemical names in **Table 2**). We evaluated compound toxicity by testing cell survival following 24 h incubation with 10 μM of each compound using crystal violet staining (**Figure 1B**). Only H16 has led to a significant decrease in cell viability and therefore not used for further analyses.

**Table 2:** Hits and analogs.

ID	Chemical name
**Hits**	
H1	5-Benzyl-2-[(2-chlorophenyl)imino]-1,3-thiazolidin-4-one
H2	5-Butyl-3-{[2-(4-morpholinyl)ethyl]thio}-5H-[1,2,4]triazino[5,6-b]indole
H3	2-Phenyl-*N*′-({5-[3-(trifluoromethyl)phenyl]-2-furyl}methylene)acetohydrazide
H4	1-{3-[(4-Chlorobenzyl)oxy]phenyl}ethanone
H5	1-Allyl-2-(3,4,5-trimethoxyphenyl)-1H-benzimidazole
H6	7-(Difluoromethyl)-*N*-[2-(4-morpholinyl)ethyl]-5-phenylpyrazolo[1,5-a]pyrimidine-3-carboxamide
H7	1-Phenyl-4-[4-(2-thienylcarbonyl)-1-piperazinyl]phthalazine
H8	4-[5-(3-Methylphenoxy)pentyl]morpholine
H9	1-(2-Fluorophenyl)-4-(phenylacetyl)piperazine
H10	2-{[2-Oxo-2-(1-piperidinyl)ethyl]thio}-4-phenyl-6-(trifluoromethyl)pyrimidine
H11	*N*-[2-(Phenylthio)cyclohexyl]benzenesulfonamide
H12	1-{[3-(Benzyloxy)phenyl]carbonothioyl}-4-methylpiperazine
H13	5-Phenyl-*N*-(2-thienylmethyl)-7-(trifluoromethyl)pyrazolo[1,5-a]pyrimidine-2-carboxamide
H14	2-(2,3-Dihydro-9H-imidazo[1,2-a]benzimidazol-9-yl)-1-(4-hydroxyphenyl)ethanone hydrobromide
H15	1-Allyl-2-(2-phenylvinyl)-1H-benzimidazole
H16	2-[(2,5-Dimethoxyphenyl)diazenyl]-1-Methyl-1H-benzimidazole
H17	2-[(2,6-Dimethyl-1-piperidinyl)carbonyl]-7-methyl-5-phenylpyrazolo[1,5-a]pyrimidine
H18	2-(4-Morpholinylmethyl)-1-(1-naphthylmethyl)-1H-benzimidazole
H19	2-(Benzylthio)-*N*-cyclopentylbenzamide
H20	5-Isopropyl-*N*-methyl-3-phenylpyrazolo[1,5-a]pyrimidin-7-amine
**Analogs**	
H8-1	4-[5-(3,5-Dimethylphenoxy)pentyl]morpholine
H8-2	4-[5-(3,4-Dimethylphenoxy)pentyl]morpholine
H8-3	4-[6-(3-Methylphenoxy)hexyl]morpholine
H8-4	4-[4-(3-Methylphenoxy)butyl]morpholine
H8-5	4-[4-(3,4-Dimethylphenoxy)butyl]morpholine
H8-6	4-[5-(3-Methoxyphenoxy)pentyl]morpholine
H8-7	4-[5-(3-Chlorophenoxy)pentyl]morpholine
H8-8	1-[5-(2-Fluorophenoxy)pentyl]-4-methylpiperazine

Next, we set to find putative targets of the identified hits by searching the Scifinder^®^ database for structure similarities between the hits and other compounds with known targets/signaling pathways. Six hits were mapped to three putative targets/pathways: Shh, S1R, and 11β-hydroxysteroid dehydrogenase type1 (11β-HSD1). We further tested them for their ability to enhance mitochondrial health using JC-1 fluorescence staining. Based on membrane potential, JC-1 red and green emissions serve as proxy of intact and damaged mitochondria, respectively. Treatments with hits H8 (putative target: S1R), H15 (putative target: Shh), and H17 (putative target: 11β-HSD1) have led to increased level of intact mitochondria, while H9 and H17 treatment also decreased the level of damaged mitochondria (**Figure 1C**). To set the ground for further drug development we sought to confirm the predicted anomaly of Shh and S1R functions in eIF2B5-mutant primary MEFs.

### Sonic Hedgehog (Shh) Pathway Regulation Is Impaired in Eif2b5^R132H/R132H^ Mutant Mice

**FIGURE 2:**
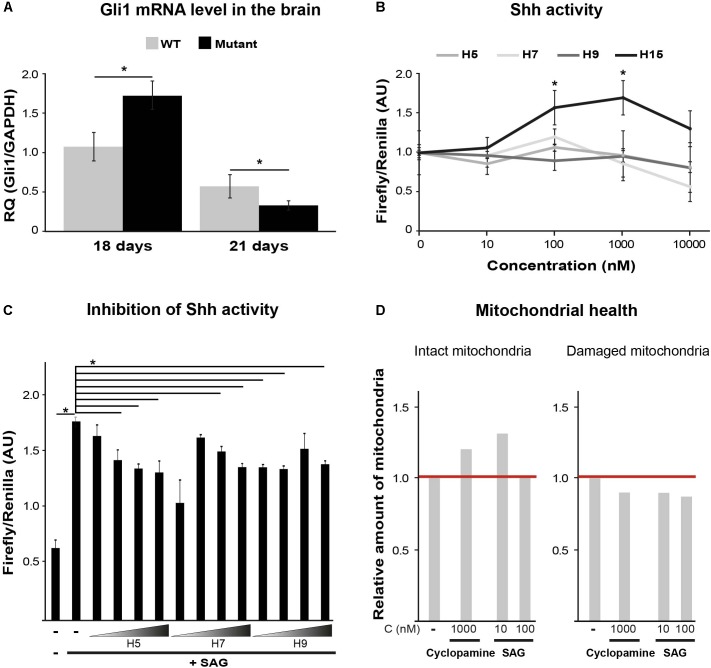
Shh pathway target validation. **(A)** RT-qPCR of Gli1mRNA using total RNA extracted from the left hemisphere of p18 and p21 WT and Mutant mice brains. Shown is relative quantity (RQ) of Gli1/Gapdh following ΔΔCT analysis ± SD. ^∗^*p* < 0.02. **(B)** Shh activity assayed in Shh-lightII cells incubated or not for 24 h with the indicated compounds at the indicated concentrations. Shown is Firefly/Renilla luciferase activity compared to untreated cells ± SD. ^∗^*p* < 0.015. **(C)** Shh activity assayed in Shh-lightII cells incubated or not for 24 h with 0.01, 0.1, 1, and 10 μM of the indicated compounds in the presence of the Shh activator SAG at 50 nM. Shown is average Firefly/Renilla luciferase activity compared to SAG-treated cells ± SD. ^∗^*p* < 0.04. **(D)** Mut MEFs incubated or not for 24 h with the indicated compounds, followed by Hoechst and JC1 staining and analysis for intact and damaged mitochondria as described in **Figure 1C**.

Sonic hedgehog is an important morphogen for central nervous system patterning during development. Among its non-patterning functions is proliferation of oligodendrocytes precursors and their further differentiation (Fuccillo et al., 2006). While Shh activity increases during initial stages of brain development to induce oligodendrocyte proliferation and maturation, later on it decreases to allow complete differentiation and myelination (Bouslama-Oueghlani et al., 2012). Shh signaling is also involved in enhanced mitochondrial health (Wu et al., 2009; Malhotra et al., 2016). To assess the involvement of Shh pathway in VWM disease, we measured by RT-qPCR the mRNA level of *Gli1*, a Shh target gene. We used brain extracts of WT and Eif2b5^R132H/R132H^ mutant mice at postnatal ages of P18 and P21, the peak of oligodendrocyte differentiation and myelin formation in mice (Verity and Campagnoni, 1988). The normal expected decrease between P18 and P21 observed in WT was more prominent in mutants. Specifically, mutants showed significantly higher and lower levels at P18 and P21, respectively, compared to WT controls (**Figure 2A**). Our putative target identification analysis suggested Shh signaling pathway for four hits (H5, H7, H9, and H15). To test this prediction, we used Shh-lightII cells, which enable a read-out of Shh activation. These cells stably express *Renilla–Luciferase* for normalization purposes and an inducible *Firefly–Luciferase* reporter gene under the control of Gli1 promoter (Taipale et al., 2000). We found that only H15 was able to activate Shh signaling pathway, as it led to increased *Firefly/Renilla luciferase* expression in a concentration-dependent manner (**Figure 2B**). Therefore, we concluded that H15 is a putative Shh agonist. To test if the rest of the compounds affect Shh pathway as antagonists, we tested their ability to inhibit the activity of a known potent *Smoothened* agonist (SAG) (Chen et al., 2002). H5 and H7 inhibited the SAG-induced Shh activity in a concentration-dependent manner, confirming their involvement in this pathway as antagonists (**Figure 2C**). To confirm the ability of both Shh agonists and antagonists to improve mitochondrial health, we tested the effect of cyclopamine (a known Shh antagonist) and SAG (agonist) on JC-1 staining. Interestingly, both SAG and cyclopamine led to beneficial effects on mitochondrial health (**Figure 2D**).

### Sigma-1-Receptor (S1R) Putative Target: Relevance and Efficacy

#### Confirmation of H8 Binding to S1R

**FIGURE 3:**
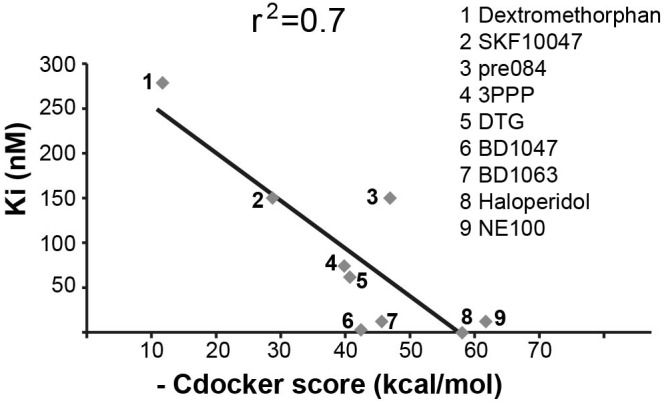
Correlation between experimentally determined Ki values and calculated docking scores. Correlation (*r*^2^ = 0.7) between known Ki values and CDOCKER scores (kcal/mol) for known S1R binders, as indicated. Higher CDOCKER values correspond to better binding energies.

Sigma-1-Receptor is a transmembrane chaperone protein located at the ER–mitochondria interface, where it regulates their cross talk and function to promote cellular survival. Among many functions, it ensures Ca^2+^ signaling from the ER into the mitochondria by chaperoning IP3 receptor (Hayashi and Su, 2007). S1R role is important for multiple cellular scenarios including astrocytes activation and oligodendrocyte proliferation, differentiation, and myelin production (Demerens et al., 1999; Lisak et al., 2014; Zhang et al., 2015). As VWM disease is associated with mitochondria malfunction (Elroy-Stein, 2017; Raini et al., 2017) and since the hallmark of the pathology is related to impaired astrocytes activation, oligodendrocytes differentiation, and myelin formation (Schiffmann and Elroy-Stein, 2006; Dooves et al., 2016), we deduced that S1R might be involved in the disease. Moreover, given the similarity of H8 structure to a known S1R ligand (Lan et al., 2014), we reasoned that H8 may also be an S1R ligand. To test this possibility, we first computationally checked whether H8 is a potential binder of S1R by docking simulations using the CDOCKER program (Wu et al., 2003) as implemented in BIOVIA’s Discovery Studio (2016). Initially, we tested whether CDOCKER can reliably reproduce the ligand-binding mode of a known binder, using the crystal structure of S1R in complex with *N*-(1-benzylpiperidin-4-yl)-4-iodobenzamide (code 5HK2 in the PDB protein data bank). Indeed, the lowest energy-binding mode obtained with CDOCKER had a root mean square value (RMSD) of 0.8 Å with respect to the crystal structure, suggesting that the program is appropriate for this study. Next, nine S1R binders with known ligand–protein binding Ki values retrieved from PubChem^1^ were docked into the S1R structure. The scores of the top ranked pose of each ligand correlated with the experimental Ki values and a significant correlation with *r*^2^ = 0.7 was obtained (**Figure 3**). Next, hit H8 docked into this structure got a score of 49.38 kcal/mol, which falls within the range of scores for the known binders. To confirm the binding of hit H8 to S1R, we employed an *in vitro* competitive displacement-binding assay using the known S1R binder [^3^H]-haloperidol (Ganapathy et al., 1999). This test revealed that 10 μM of H8 inhibited 92% of Haloperidol binding, confirming that H8 (see chemical name in **Table 2**) is a direct S1R binder.

### Decreased S1R Expression in Eif2b5^R132H/R132H^ Mutant Mice

**FIGURE 4:**
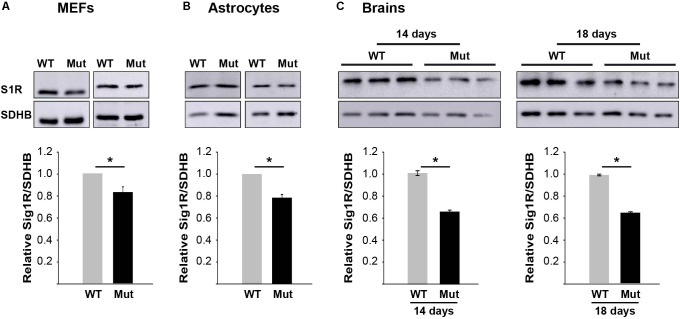
S1R protein level is lower in mutants. Equal amounts of total protein of primary MEFs **(A)**, primary astrocytes **(B)**, or brains at postnatal age of P14 and P18 **(C)** isolated from WT and mutant mice were subjected to immunoblot analysis using antibodies specific for S1R and SDHB as a marker for mitochondria content. Shown are representative blots (top) and calculated S1R/SDHB ratio ± SEM of three to six independent experiments (bottom). ^∗^*p* = 0.02 (MEFs), ^∗^*p* = 0.01 (astrocytes), ^∗^*p* = 0.048 (p14 brains), and ^∗^*p* = 0.003 (p18 brains).

To set the ground for further drug development toward S1R as a relevant target we first tested if there is any VWM-related abnormality in S1R expression level. We recently discovered that primary MEFs and astrocytes isolated from Eif2b5^R132H/R132H^ mice suffer from oxidative respiration defects, leading to increased mitochondrial biogenesis for compensation purposes to meet energetic needs (Raini et al., 2017). It was therefore important to test S1R protein level per mitochondria content. For this purpose, we tested the ratio between S1R protein level and the level of nuclear-encoded SDHB protein, a component of the mitochondrial electron transfer chain (ETC) complex. Western blot analysis revealed that primary MEFs and astrocytes isolated from mutant mice express 17 and 22% lower S1R/SDHB protein ratio compared to WT MEFs and astrocytes, respectively (**Figures 4A,B**). Brain extracts of mice at postnatal ages of P14–P18 showed S1R/SDHB levels lower by 34–35% compared to WT controls (**Figure 4C**).

### Effect of S1R Binders on Abundance and Function of Mitochondria in Eif2b5^R132H/R132H^ MEFs and Astrocytes

**FIGURE 5:**
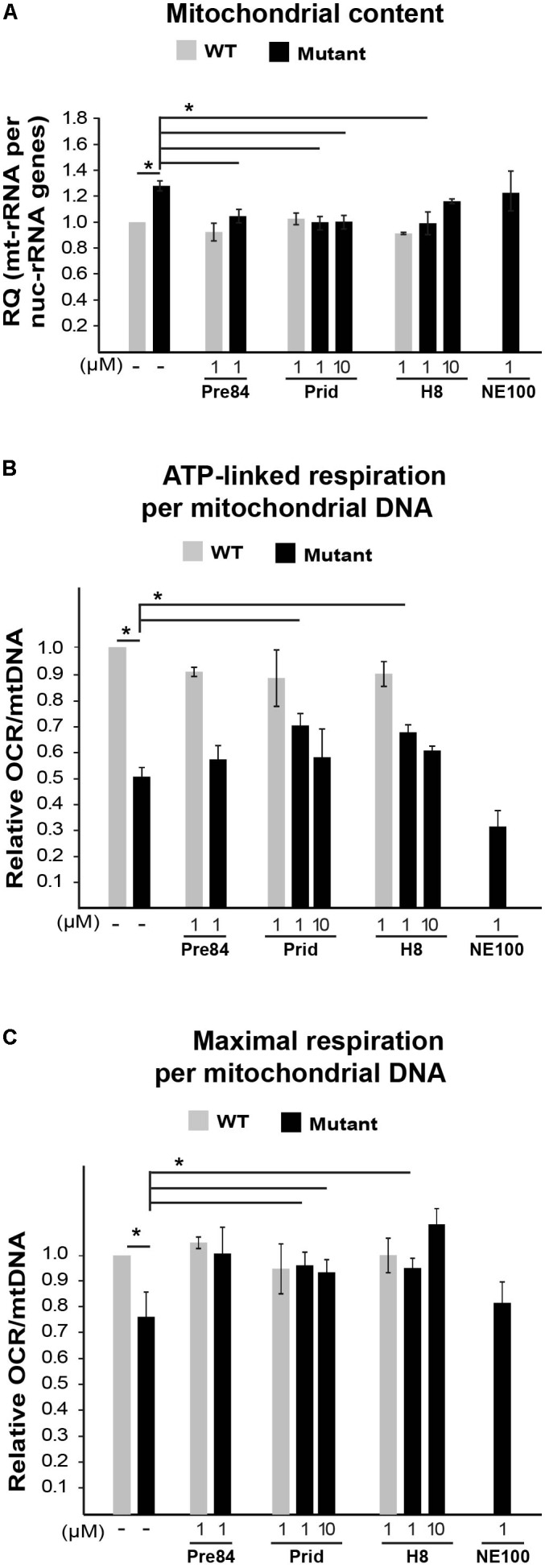
Effect of S1R binders on mitochondrial abundance and performance in mutant MEFs. **(A)** qPCR analysis of mitochondrial 12S rRNA and nuclear 18S rRNA genes in WT (gray) and Mut (black) MEFs incubated or not with the indicated compounds for 6 h. Shown is average RQ of mtDNA per nucDNA values of two to seven independent experiments normalized to untreated WT ± SEM. ^∗^*p* < 0.03. **(B,C)** Oxygen consumption rate (OCR) in WT and Mut MEFs incubated or not with the indicated compounds for 6 h. Values of ATP-linked respiration **(B)** and maximal respiration **(C)** analyzed and normalized to mtDNA per nucDNA values obtained in **(A)**. Shown are average values normalized to untreated WT MEFs ± SEM of six replicates in a representative experiment of three independent experiments. ^∗^*p* < 0.05.)

**FIGURE 6:**
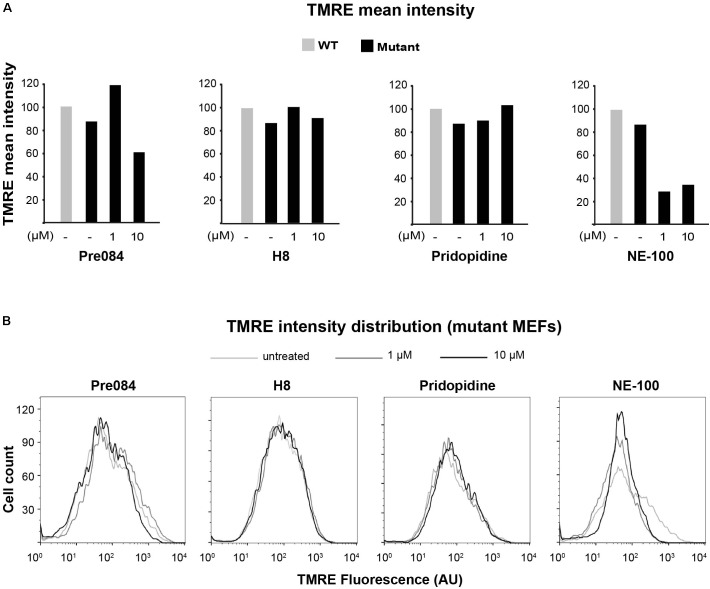
Effect of S1R binders on mitochondria membrane potential in mutant MEFs. Flow cytometry analysis of WT and Mut MEFs incubated or not with the indicateds for 6 h and stained with TMRE. Shown is TMRE mean intensity **(A)** and TMRE intensity distribution **(B)** of a representative experiment.

To further analyze the involvement of S1R in VWM disease, we tested the effect of Pre084 and NE-100, known S1R agonist and antagonist, respectively, on mitochondria content in primary MEFs isolated from Eif2b5^R132H/R132H^ mice. In addition to hit H8, the effect of pridopidine was also tested due to its high affinity to S1R (Sahlholm et al., 2015) and its beneficial effects on a mouse model of Huntington disease, another neurodegenerative pathology (Ryskamp et al., 2017). Incubation for 6 h with 1 μM of Pre084 corrected the abnormal high mitochondria content in mutant MEFs and brought it closer to WT level. A similar effect was observed for pridopidine and H8, suggesting that H8 functions as a S1R agonist (**Figure 5A**). In contrast to these results, NE-100 did not decrease the mitochondria content of mutant MEFs, i.e., did not correct their anomaly. The lack of any negative effect on survival of mutant MEFs upon incubation with as high as 30 μM of Pre084, pridopidine, or H8 (**Table 3**), ruled out decreased mitochondria content due to a toxic effect. To verify that decreased mitochondria content is due to a repair effect, we stained the cells with TMRE, a positively charged fluorescent dye specific for active mitochondria with membrane potential above a certain threshold (Crowley et al., 2016). While incubation of mutant MEFs for 6 h with 1 μM of NE-100 dramatically decreased TMRE staining, similar concentration of Pre084, H8, and pridopidine did not lead to such a toxic outcome (**Figure 6**). Moreover, 1 μM of H8 and 10 μM of pridopidine increased TMRE staining and brought it to the level of untreated WT cells. The lower mitochondria content in S1R agonists-treated MEFs (**Figure 5A**) is consistent with the idea that S1R agonists positively affect membrane potential by elevating mitochondrial Ca^2+^ concentration, resulting in upregulation of the entire oxidative phosphorylation machinery for more efficient ETC activity and higher ATP synthesis (Brookes et al., 2004). To test if mitochondria content normalization is a result of enhanced oxidative respiration, we measured oxygen consumption rate (OCR). While NE-100 prompted a negative effect, S1R agonists did not elicit any effect on respiration per cell (not shown). However, given that H8 and pridopidine lead to decreased mitochondria content and increased mitochondrial membrane potential (**Figures 5**, **6**), we normalized the OCR data to mitochondrial DNA content and revealed a statistically significant increase in ATP-linked and maximal respiration rates per mitochondria in mutant MEFs following incubation with 1 μM H8 or pridopidine (**Figures 5B,C**).

**Table 3:** Effect of compounds on cell survival.

			Survival (%) ± SEM		
		μM	Mut MEFs	WT astrocytes	Mut astrocytes
Sig1R binders	pre084	0.1	100 ± 2.1		
		1	101 ± 3.5	114 ± 7.9	97 ± 0.5
		10	99 ± 1.5	95 ± 2.3	93 ± 5.1
		20	97 ± 2.1		
		30	105 ± 1.9	82 ± 1.9 (*p* = 8.3*E*–04)	76 ± 4.9 (*p* = 0.03)
		50	80 ± 2.8 (*p* = 0.01)		
		100	57 ± 2.2 (p = 1.5*E*–05)		
	Pridopidine	0.1	102 ± 1.7		
		1	103 ± 1.3	103 ± 3.5	97 ± 2.7
		10	98 ± 1.6	101 ± 2.2	92 ± 2.8
		20	99 ± 0.64		
		30	99 ± 3.1	85 ± 4.3 (*p* = 0.02)	97 ± 5.0
		50	97 ± 1.2		
		100	91 ± 2.4 (*p* = 0.03)		
	^∗^H8	0.1	104 ± 4.6		
		1	102 ± 3.3	132 ± 10.7	108 ± 0.6
		10	99 ± 2.6	102 ± 3.8	93 ± 2.1
		20			
		30	105 ± 13	99 ± 3.2	92 ± 1.6 (*p* = 0.14)
		50			
		100			
	NE100	0.1	99 ± 2.1	98 ± 1.8	
		1	98 ± 2.2	86 ± 1.1 (*p* = 1.5*E*–5)	94 ± 4.7
		10	88 ± 3.5 (*p* = 0.017)	79 ± 2.2 (*p* = 1.6*E*–4)	82 ± 4.3 (*p* = 0.01)
		30			65 ± 2.2 (*p* = 6.0*E*–06)
H8 analogs	H8-1	10	104 ± 2.7		
		20	103 ± 4.7		
		30	97 ± 3.9		
	H8-2	10	92 ± 4.2		
		20	94 ± 8.8		
		30	95 ± 13.1		
	H8-3	10	100 ± 7.2		
		20	96 ± 4.1		
		30	88 ± 4.5 (*p* = 0.09)		
	H8-4	10	92 ± 8.5		
		20	95 ± 4.2		
		30	98 ± 5.4		
	H8-5	10	92 ± 1.7		
		20	100 ± 4.4		
		30	98 ± 13.8		
	H8-6	10	97 ± 5.1		
		20	92 ± 1.1		
		30	93 ± 5.2		
	H8-7	10	98 ± 5.1		
		20	91 ± 4.7		
		30	88 ± 9.7 (*p* = 0.44)		
	H8-8	10	101 ± 5.6		
		20	99 ± 11		
		30	90 ± 3.5		
Hedgehog modulators	SAG	0.01	98 ± 2.1		
		0.05	92 ± 3.2		
		0.1	95 ± 2.1		
		1	68 ± 2.7 (*p* = 1.9*E*–05)		
	Cyclopamine	0.1	92 ± 2.5		
		1	95 ± 4.1		
		10	79 ± 1.4 (*p* = 0.02)		

*Primary MEFs and astrocytes isolated from Eif2b^R132H/R132H^ (Mut) or WT mice were incubated for 24 h with the indicated compounds/concentrations followed by Crystal Violet staining. Cell survival of untreated cells was set to 100%. Shown is % cell survival ± SEM of at least three repeats. Toxicity refers to survival rate below 90% (marked gray, p-values indicated).*

**FIGURE 7:**
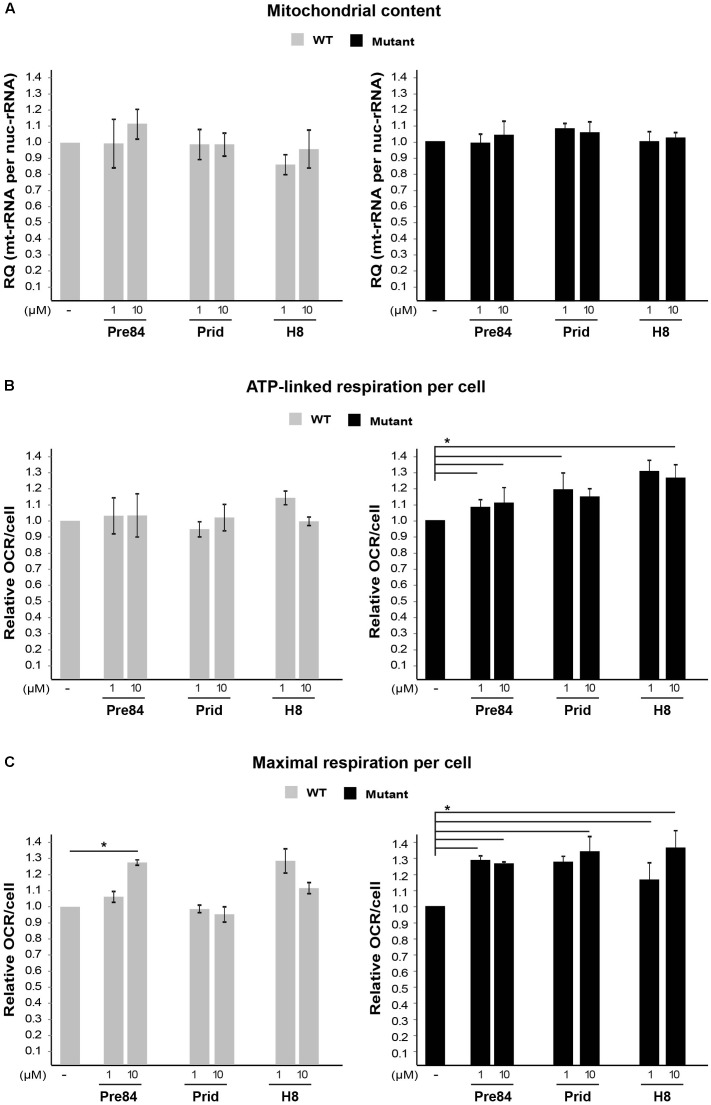
Effect of S1R binders on mitochondria abundance and performance in astrocytes. **(A)** qPCR analysis of DNA encoding mitochondrial 12S rRNA and nuclear 18S rRNA in WT (gray) and Mut (black) primary astrocytes incubated or not with the indicated compounds for 6 h. Shown is average RQ of mtDNA per nucDNA values of two to six independent experiments normalized to their untreated controls ± SEM. **(B,C)** OCR in WT and Mut astrocytes incubated or not with the indicated compounds for 6 h. Values of ATP-linked respiration **(B)** and maximal respiration **(C)** analyzed and normalized to cell number. Shown are average values normalized to untreated cells ± SEM of six replicates in a representative experiment from four independent experiments. ^∗^*p* < 0.05.

Next, due to the specific involvement of astrocytes in VWM disease, it was important to test the effect of S1R agonists on primary astrocytes isolated from Eif2b5^R132H/R132H^ mutant mice. Interestingly, while 1 or 10 μM Pre-084, pridopidine, or H8 increased both ATP-linked and maximal respiration in mutant astrocytes (**Figures 7B,C**), no effect on mitochondria content per cell was observed (**Figure 7A**). This phenomenon is consistent with the beneficial outcome of S1R agonists, yet the partial correction of the anomaly in astrocytes is consistent with the high energetic requirements of these cells.

### Effect of S1R Binders on Sensitivity of Eif2b5^R132H/R132H^ MEFs and Astrocytes to ER Stress

**FIGURE 8:**
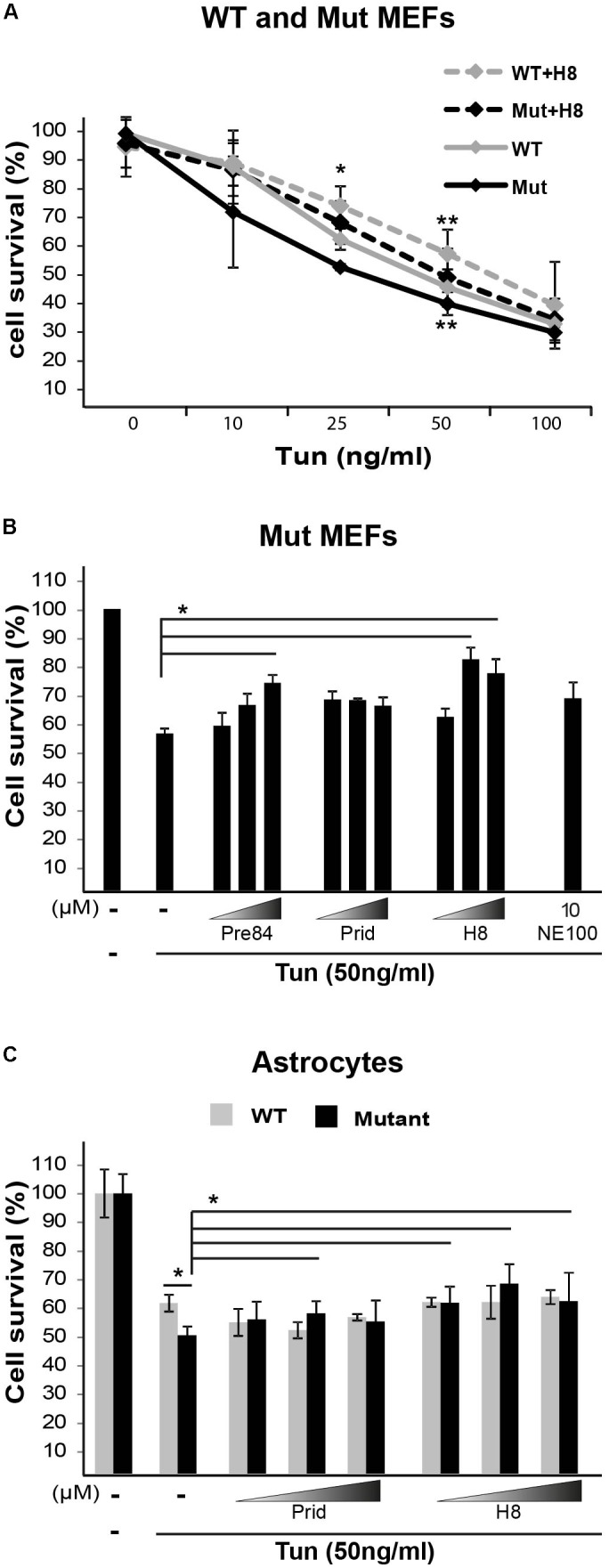
S1R binders improve cell survival upon ER stress. **(A)** WT and Mut MEFs were pretreated or not for 2 h with 20 μM H8 followed by addition of the indicated concentrations of Tunicamycin (Tun) for additional 22 h. Cell survival was determined by crystal violet staining. Shown are average values normalized to untreated controls ± SEM of three independent experiments. ^∗^*p* < 0.05 for Mut vs. WT at 25 ng/ml Tun; ^∗∗^*p* < 0.05 for WT+H8 vs. WT and for Mut+H8 vs. Mut at 50 ng/ml Tun. **(B,C)** Mut MEFs **(B)** or WT and Mut astrocytes **(C)** were pretreated or not for 2 h with the indicated compounds at the indicated concentrations (**B**: 10, 20, 30 μM; **C**: 20, 30, 50 μM), followed(addition of 50 ng/ml Tun for additional 22 h and then stained with crystal violet. Shown are average values normalized to Tun-treated cells without any compound ± SEM of three independent experiments, ^∗^*p* < 0.05.by addition of 50 ng/ml Tun for additional 22 h and then stained with crystal violet. Shown are average values normalized to Tun-treated cells without any compound ± SEM of three independent experiments, ^∗^*p* < 0.05.by addition of 50 ng/ml Tun for additional 22 h and then stained with crystal violet. Shown are average values normalized to Tun-treated cells without any compound ± SEM of three independent experiments, ^∗^*p* < 0.05.by addition of 50 ng/ml Tun for additional 22 h and then stained with crystal violet. Shown are average values normalized to Tun-treated cells without any compound ± SEM of three independent experiments, ^∗^*p* < 0.05.)

Primary fibroblasts isolated from VWM patients are hypersensitive to pharmacologically induced ER stress (Kantor et al., 2005). The hypersensitivity of mutant MEFs isolated from Eif2b5^R132H/R132H^ mice was confirmed by their lower survival rate compared to WT controls upon 22 h incubation with the ER stress agent Tunicamycin (Tun), as assayed by crystal violet staining (**Figure 8A**). Since S1R is involved in ER function, we sought to test whether hit H8 affects cell survival under ER stress conditions. Pre-treatment with 20 μM H8 for 2 h followed by its presence throughout 22 h incubation with Tun increased the survival of mutant MEFs to the same rate of Tun-treated WT MEFs in the absence of H8, demonstrating its ability to rescue the ER hypersensitivity of mutants (**Figure 8A**). 30 μM Pre084 was also able to rescue mutant MEFs from Tun-induced ER stress-mediated cell death (**Figure 8B**). Importantly, primary astrocytes isolated from Eif2b5^R132H/R132H^ mice exhibited hypersensitivity to ER stress compared to astrocytes isolated from WT control mice. While S1R agonists did not rescue WT astrocytes, 30 μM pridopidine and 20–50 μM H8 increased the survival rate of mutant astrocytes under induced ER stress conditions (**Figure 8C**).

### H8 Analogs

**FIGURE 9:**
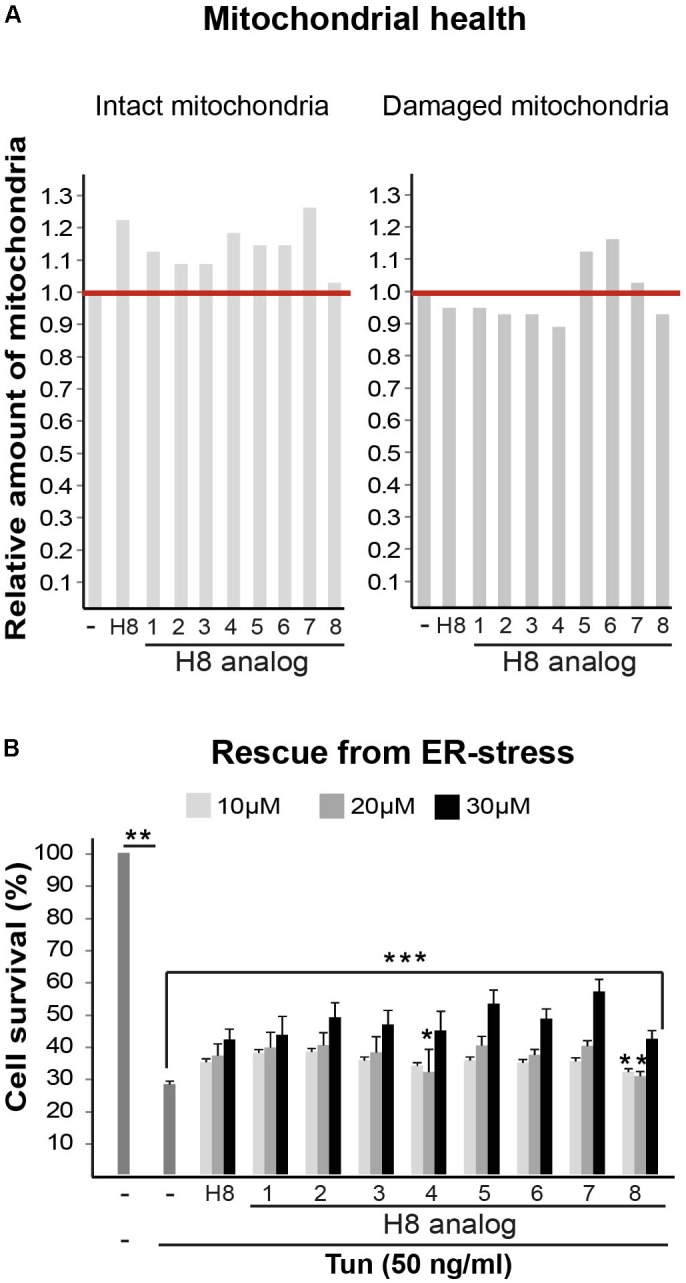
H8 analogs improve mitochondrial health and cell survival following Tunicamycin-induced ER stress. **(A)** Mut MEFs incubated or not for 24 h with 10 μM of the indicated compounds (**Table 1**), followed by Hoechst and JC1 staining and analysis for intact and damaged mitochondria as described in **Figure 1**. **(B)** Mut MEFs incubated or not with the indicated concentrations of the indicated compounds for 2 h followed by addition of 50 ng/ml Tun for additional 22 h incubation and then stained with crystal violet. Shown are average values normalized to Tun-treated cells without any compound ± SEM of three independent experiments. ^∗^not significant; ^∗∗^*p* < 0.02; ^∗∗∗^*p* < 0.04.

Taken together, the data indicate that treatment of mutant cells with S1R agonists (i.e., Pre084, pridopidine, and H8) leads to increased mitochondrial membrane potential, enhanced oxidative respiration, and increased ability of eIF2B5-mutant MEFs and astrocytes to cope with chronic ER stress. The finding that S1R expression level is low in primary MEFs, astrocytes, and brains isolated from Eif2b5^R132H/R132H^ mice suggests that S1R is a favorable potential target for the treatment of VWM disease and that compounds acting as S1R agonists such as Pre084, pridopidine, and hit H8 are plausible starting points for the development of relevant therapeutic strategy. To find a more potent starting point for drug development based on the current study, we set to identify structural analogs of H8. For this purpose, we scanned other EnamineStore and Chembridge libraries for compounds with structure similarity to that of H8. Eight similar analogs (listed in **Table 2**) were tested for their effect on mitochondrial health and cell survival following Tun-induced cell death. Five analogs elicited beneficial effect on mitochondrial health, as they increased the level of intact mitochondria while four of them also decreased the level of damaged mitochondria (**Figure 9A**). All the analogs exhibited a similar or better effect compared to H8 in the ER stress-induced cell death assay, as all increased cell survival. Analogs H8-2, H8-5, H8-6, and H8-7 displayed the most potent effect at 30 μM concentration (**Figure 9B**).

## Discussion

In the current study, we took advantage of the abnormal increased mitochondria content in Eif2b5^R132H/R132H^ primary MEFs (Raini et al., 2017), for screening of the DIVERSet^TM^-EXP library that contains 50,000 drug-like small molecules. The increased mitochondrial biogenesis, a compensatory response of mutant cells to their decreased oxidative phosphorylation, has set the rational for our approach to assay mitochondria content by single cell-based imaging analysis. We reasoned that the decrease in mitochondria content in mutants could be the outcome of either toxic or healing scenario initiated by the tested compound. While the assay is simple, it was associated with a major challenge. First, there is only 20–50% increase in mitochondria content in mutant compared to WT MEFs. Second, cellular metabolism and mitochondrial biogenesis are extremely sensitive to multiple variables related to experimental conditions, some of which are beyond tight control. These limitations enforced us to avoid expensive high-throughput screening. Instead, we used rational computational approaches; first for the selection of the optimal screening library, followed by its clustering based on structure similarities within its compounds. This strategy enabled us to minimize the number of compounds tested, thereby allowing for multiple repetitions of biological testing. Thus, we tested only 500 representative compounds in the first round and ∼500 most relevant compounds (consisting the members of the relevant positive clusters) in the second round, instead of the entire 50,000 compounds library. Surprisingly, only one hit (H16) decreased mitochondria content in mutant MEFs due to a toxic effect, while all other hit compounds reduced the need for mitochondrial biogenesis due to beneficial effects on mitochondrial health (**Figure 1**). Subsequent analysis of structural similarities between our primary hits and other compounds with known targets afforded the identification of three putative cellular pathways/targets: 11β-HSD1, Shh, and S1R. This finding is significant by itself, as it is the first time these cellular components are mentioned in the context of VWM disease.

11β-Hydroxysteroid dehydrogenase type 1 enzyme is widely expressed throughout the central nervous system. It regenerates active glucocorticoids from their inactive 11-keto derivatives and possesses 7β-hydroxycholesterol dehydrogenases activity involved in oxysterol metabolism (Wyrwoll et al., 2011). The involvement of this enzyme in VWM disease is proposed since 7-ketocholesterol and 7β-hydroxycholesterol cause phenotypic characteristics associated with the disease. These include ER stress, impaired mitochondrial function and increased ROS production which lead to apoptosis and autophagy in murine oligodendrocytes (Nury et al., 2015). The actual implication of 11β-HSD1 in VWM pathology is yet to be determined.

Shh signaling pathway is involved in many cellular contexts and molecular mechanisms. The canonical scenario involves cell-surface binding of a ligand, leading to the formation of repressor or activator forms of the GLI/CI family members of zinc-finger transcription factors (Fuccillo et al., 2006). Shh activity is required for oligodendrocytes generation, maturation, and myelin formation during early brain development. The abnormal high and low Gli1 mRNA level at P18 and P21, respectively, in the brains of mutant mice, indicate abnormal Shh signaling due to the mutation in eIF2B during the peak of myelin formation (**Figure 2A**). This important observation, pointing at impaired programming of Shh pathway in mutants, is consistent with our previous finding related to delayed waves of gene expression during early brain development in Eif2b5^R132H/R132H^ mouse model (Marom et al., 2011). The impact of Shh signaling modulation on mitochondrial health in mutant MEFs (**Figure 2D**) further supports the relevance of Shh pathway to VWM pathology. Moreover, Shh signaling is required for the maintenance of endogenous adult neural stem cells (NSCs) which serve as a source for remyelination in the adult brain (Fuccillo et al., 2006). Interestingly, Samanta et al. (2015) reported that Gli1 inhibition enhances NSCs recruitment to demyelinated lesions and increases their differentiation into oligodendrocytes. Remarkably, the small molecule GANT61 successfully inhibits Gli1 and improves remyelination in an experimental animal model for multiple sclerosis (Samanta et al., 2015). Taken together, pharmacological targeting of the Shh signaling pathway may serve as a new therapeutic opportunity for the treatment of VWM disease. Demyelination is a hallmark of VWM pathology, thus additional work is required for characterization of Shh signaling pathway role in the etiology of the disease. Further effort will be necessary for elucidating the specific binding targets of Shh-related compounds identified in this study (**Figure 2**). Once elucidated, compounds H5, H7, H9, and H15 may be attractive candidates for further drug development, for the treatment of VWM and additional demyelinating disorders.

Sigma-1-Receptor plays important roles in many physiological functions and known as a pluripotent modulator in living systems. While it resides in the mitochondria-associated ER membrane (MAM), it can also translocate to the plasma membrane to interact with ion channels and other receptors to affect their function, and interact with cytoplasmic soluble proteins. Furthermore, S1R can translocate to the nuclear envelope to recruit chromatin-remodeling factors and thereby affect the transcription of genes. S1R promotes cell survival under physiological stress conditions by regulating ER-mitochondria Ca^2+^ flux, signaling for antioxidants expression and attenuating free radicals damage (Su et al., 2016). The increase in ATP-linked and maximal respiration rates by S1R agonists treatment of mutant cells (**Figures 5**, **7**) clearly indicates the beneficial effect of S1R activation in the context of VWM pathology. Not only that it enhances mitochondrial function most probably by modulating Ca^2+^ signaling, it also enhances the ability of mutant cells to cope with chronic ER stress (**Figure 8**), supposedly by activating the mitochondrion–ER–nucleus signaling axis, as previously reported, to counteract ER stress (Mori et al., 2013).

Due to the wide range of S1R effects on cellular physiology, it is not surprising that highly sensitive brain cells are profoundly dependent on its function. This includes astrocytes activation (Zhang et al., 2015), axon outgrowth (Li et al., 2017), oligodendrocyte proliferation, differentiation, and myelin production (Demerens et al., 1999; Lisak et al., 2014). Interestingly, S1R knockout mice display locomotor deficits associated with muscle weakness, axonal degeneration, and motor neuron loss because of affected intracellular calcium signaling, ER stress, and defects in mitochondrial dynamics due to dysfunctional ER–mitochondria crosstalk (Bernard-Marissal et al., 2015). The intense impact of S1R function on the central nervous system explains its involvement in multiple neurological disorders including Alzheimer’s disease, major depressive disorders, and schizophrenia (Nguyen et al., 2017). Therefore, S1R activation is highlighted as neuroprotective and neurorestorative for various disorders (Ruscher and Wieloch, 2015; Li et al., 2017). The current work demonstrates for the first time the beneficial effect of S1R agonists on eIF2B-mutant cells. Together with the low S1R expression in mutants compared to controls (**Figure 4**), this study marks S1R as a strong therapeutic target for the development of a treatment for VWM disease. The cellular effect of several known S1R agonist molecules was already partially evaluated and classified (Maurice and Su, 2009). In the current study, we identified an additional S1R agonist, i.e., 4-[5-(3-methylphenoxy)pentyl]morpholine, and assayed eight analogs of this compound (**Figure 9** and **Table 2**) for their effect on mitochondrial function. These compounds serve as promising drug-like candidates for the development of a treatment for VWM disease as well as for other relevant central nervous system pathologies.

## Author Contributions

AA designed and performed experiments, interpreted data, organized and created figures, and helped with manuscript preparation. RS-E contributed to image-based screening and analyses. MH performed and interpreted astrocytes experiments, contributed to figures preparation, and manuscript editing. YG performed rational computational approaches. HS designed and guided computational approaches, critically discussed the data, and contributed to manuscript preparation. OE-S initiated, designed, and conducted the project, critically discussed the data, and wrote the manuscript.

## Conflict of Interest Statement

The authors declare that the research was conducted in the absence of any commercial or financial relationships that could be construed as a potential conflict of interest.

## Footnotes

1 http://pubchem.ncbi.nlm.nih.gov
